# Optimization of Extraction and HPLC–MS/MS Profiling of Phenolic Compounds from Red Grape Seed Extracts Using Conventional and Deep Eutectic Solvents

**DOI:** 10.3390/antiox11081595

**Published:** 2022-08-18

**Authors:** Nevena Dabetic, Vanja Todorovic, Andjelija Malenovic, Sladjana Sobajic, Bojan Markovic

**Affiliations:** 1 Department of Bromatology, Faculty of Pharmacy, University of Belgrade, Vojvode Stepe 450, 11221 Belgrade, Serbia; 2 Department of Drug Analysis, Faculty of Pharmacy, University of Belgrade, Vojvode Stepe 450, 11221 Belgrade, Serbia; 3 Department of Pharmaceutical Chemistry, Faculty of Pharmacy, University of Belgrade, Vojvode Stepe 450, 11221 Belgrade, Serbia

**Keywords:** grape seed, natural deep eutectic solvent (NADES), Box–Behnken design, validation, catechins, antioxidant tests

## Abstract

Winemaking generates large quantities of grape waste consisting of seeds, skin and stalks. Given that grape seeds are a rich source of different bioactive compounds, the main goal of this research was to optimize grape seed phenol extraction using a Box–Behnken design. The following conditions were derived from the optimization process: sample:solvent ratio of 1:10 *w*/*v*, extraction time of 30 min and extraction temperature of 50 °C. In addition, a sustainable (green) approach for obtaining extracts was developed by comparing choline chloride:citric acid-ChCit (natural deep eutectic solvent (NADES)) and ethanol extraction methods with respect to phenol profiles and antioxidant activity. This study was conducted on seeds from eight different red grape varieties. Phenolic acids, flavan-3-ols and procyanidins were characterized using HPLC–MS/MS, and the concentration of procyanidin B1 was above 1 mg/g of dry weight in all analyzed samples. The contents of all phenol classes and antioxidant activities were found to not differ significantly between the solvents, but NADES was found to offer valuable advantages. Importantly, ChCit showed a strong affinity toward procyanidins and a strong correlation between antioxidant activity and quantified phenolic compounds.

## 1. Introduction

Approximately 75% of the entire grape production industry is geared towards winemaking. Wine production is one of the most important agricultural activities globally and is associated with the generation of a large amount of solid organic waste [1]. Along with the rapid development of the wine industry in the past few decades, there has been a constant increase in grape pomace production, causing a serious economic and environmental problem. Sustainable wine production should be particularly focused on reducing the quantity of waste through potential recycling and reuse. Pomace generated in the wine industry is still underutilized; only a small percentage is exploited for animal feed, as compost and, on a small scale, for thermal insulation in building construction [2,3,4,5]. The amount of pomace is dependent on the grape variety, pressing process, and fermentation steps, but in general, it represents approximately 20–30% of the original grape weight and consists of the remaining skin, seeds and stalks [2]. In recent years, numerous studies have investigated the biological activity of grape pomace generated by the wine industry. It has been reported that the whole pomace, as well as different parts of it, possess antioxidant, anti-inflammatory and antimicrobial activities [6,7,8,9].

Grape seeds are a rich source of food and non-food applicable bioactive molecules, mainly phenols. These compounds have been successfully extracted from the seeds using different extraction approaches [10,11,12]. Currently, manufacturing processes that do not involve some type of natural product extraction method are largely non-existent across the food, perfume, cosmetic, biofuel and pharmaceutical industries [13]. Traditional extraction methods have many disadvantages, and as modern society requires environmentally friendly procedures, green extraction is promoted as a very important tool in green chemistry and overall sustainable product development [14]. New technologies such as ultrasound-assisted extraction, microwave-assisted extraction and supercritical fluid extraction have enormous “green” potential, as they provide higher extraction yields and higher-quality extracts while reducing or eliminating toxic solvents [15]. The latter is especially important since the major drawback of conventional extraction processes is the use of extremely large amounts of organic solvents [13].

Due to properties such as non-flammability, biodegradability and non-volatility, natural deep eutectic solvents (NADES) attract a lot of attention as green alternatives. In addition to environmental concerns, solvents should be chosen to assure the highest extraction efficiency. Since phenols are susceptible to oxidation, and some of them with interflavan linkages are acidic labile, extraction solvent characteristics such as polarity and pH have a strong influence on their stability and, consequently, on phenolic yield [16]. The strong impact of the solvent used for extraction on phenol content/composition of grape seeds has been previously reported [17,18]. For instance, Mandic et al. investigated extraction efficiency of ethanol and ethyl acetate for polyphenols from grape seeds. They observed that, although ethanol resulted in higher yields, ethyl acetate gave extracts with higher total soluble polyphenols and flavan-3-ol content [17]. Ethyl acetate, acetone and methanol have proven highly efficient in phenol extraction, but they are not preferable for food applications. Therefore, much subsequent research has focused on exploring environmentally friendly solvents. Another study that explored extraction efficiency of water and ethanol in different concentrations reported 50% aqueous ethanol as the most effective for extracting phenols from grape seeds [18]. Felhi et al. examined solvents of increasing polarity; hexane, dichloromethane, ethyl acetate, acetone, ethanol and water. The maximum phenolics were found in the ethanol, ethyl acetate and acetone grape seed extracts, respectively [19]. An appropriate solvent should limit phenol degradation and thus support the most-efficient phenol extraction method. NADES are mainly mixtures of a charged acceptor and a charged hydrogen donor that bind to each other with hydrogen bonds [20]. A hydrogen bond acceptor (HBA) is usually an organic salt (quaternary ammonium or phosphonium salt), while different amines, sugars, polyalcohols and carboxylic acids can be used as the hydrogen bond donor (HBD). NADES have the possibility of modifying their structure, and thus changing their physicochemical properties, which expands the range of their effectiveness and applications [14]. This is particularly important because of the structural diversity of plant phenols and the consequent impossibility to select an extraction solvent suitable for all plant phenolics. Importantly, the number of combinations of substances that can create NADES is very high. Therefore, it is possible to design a solvent for a certain purpose by mixing different compounds and in different molar ratios. A recent study examined the efficiency of nine differently composed NADES in extracting phenolic acids, flavonols and flavan-3-ols from muscadine grape skin and seeds. Choline chloride:levulinic acid:ethylene glycol (molar ratio 1:1:2) was the most effective in extracting ellagic acid, while another NADES (choline chloride:proline:malic acid; molar ratio 1:1:1) was more selective towards extracting catechins [21].

In addition to the extraction solvent, various extraction conditions, such as sample:solvent ratio, temperature and time can affect phenolic recovery. Phenol yield can be enhanced by a higher solvent:sample ratio, heating and prolonged time [22]. When trying to optimize any analytical method, there is the need to consider both the impact of independent factors and the interactions between them [23]. Several chemometric techniques have been applied for optimizing extraction processes in recent years. Box–Behnken design (BBD) is one of them. BBD represents a response-surface methodology that is more proficient and more powerful than other designs such as the three-level full factorial design, central composite design (CCD) and Doehlert design. In this design, the variables/factors require three levels, and their combinations are at the midpoints of edges of the process space and at the center. In BBD, there are no variable/factor combinations at the extremes, represented by the corner points and the star points that we have in CCD. The output of BBD is a quadratic model containing intercept, linear, interaction (products of two factors) and squared terms. The model terms/coefficients are assessed for their relevance and statistical significance in order to evaluate the effect of each independent variable/factor on the response. Statistical significance of model terms implies a significant effect of the corresponding factor or factor interaction on the response. Furthermore, the larger absolute value of the model term for coded factor values, the larger the expected effect on the response. A positive sign for a model term suggests an increased response with an increase of the corresponding factor and vice versa.

HPLC–MS has been widely applied to analyze complex phenolic mixtures after minor sample purification due to its higher sensitivity and selectivity [24,25]. An important factor that contributes to selectivity is the column packing. Compared to C18 and phenyl hexyl columns, Agilent pentafluorophenyl (PFP) columns have shown improved chromatographic separation for isomeric phenols sharing the same *m*/*z* values as precursor and product ions [26,27].

Considering the challenges mentioned above, the aim of this study was to optimize extraction by using an experimental design to maximize phenol yield from grape seeds. In addition, a comparison between choline chloride: citric acid (green solvent) and ethanol (conventional solvent) regarding extraction efficiency was performed. Furthermore, the phenol profile and antioxidant activity of obtained extracts were thoroughly investigated.

## 2. Materials and Methods

### 2.1. Chemicals and Reagents

Trolox (97%), 2,4,6-tris(2-pyridyl)-s-triazine) (TPTZ), 2,2ʹ-diphenyl-1-picrylhydrazyl (DPPH), 2,2ʹ-azino-bis (3-ethylbenzthiazoline-6-sulphonic acid) (ABTS) and neocuproin were purchased from Sigma-Aldrich (St. Louis, MO, USA). HPLC-grade ethanol, glacial acetic acid and hydrochloric acid were obtained from Fisher Chemicals (Fair Lawn, NJ, USA). Choline–chloride, dimethylaminocinnamaldehyde (DMAC) and formic acid were supplied by Acros Organics (Fair Lawn, New Jersey, USA). Folin–Ciocalteu reagent (FC), potassium hydroxide, sodium carbonate, ferric chloride hexahydrate and potassium peroxydisulfate were purchased from Merck (Darmstadt, Germany). Acetonitrile LC–MS-grade purchased from J.T. Baker (Gliwice, Poland) and ultra-pure water (prepared by TKA GenPure, Niederelbert, Germany) were used as solvents for mobile phase preparation. The phenolic standards used in this work were obtained as follows: malic acid, tartaric acid, gallic acid and citric acid from Acros Organics (Fair Lawn, NJ, USA); protocatechuic acid, (+) catechin, (−) epicatechin, procyanidin B1 and procyanidin B2 from Sigma Aldrich (St. Louis, MO, USA); and gallocatechin and epigallocatechin from Fluka (Buchs, Switzerland). All the standards were prepared as stock solutions in 70% ethanol and stored in darkness at 4 °C. Stock solutions were appropriately diluted in order to obtain optimal concentration ranges for making calibration curves.

### 2.2. Solvent Preparation

The NADES, choline chloride: citric acid (ChCit), was prepared by mixing quaternary ammonium salt (choline chloride) and a hydrogen bond donor (citric acid) at the respective molar ratio (2:1) with 30% water (*v*/*v*). After that, the mixture was stirred in a sealed flask for 2 h at 50 °C until a clear and homogenized transparent liquid was obtained. For the conventional solvent, acidified 70% ethanol (EtOH) was made by mixing ethanol, distilled water and acetic acid (70:29.8:0.2; *v*/*v*/*v*).

### 2.3. Plant Material and Sample Preparation

Vitis Vinifera fruits, commonly known as grapes, were harvested at their technological maturity from Milosavljevic and Matalj Vineyards, Regions 3 Morave and Negotinska krajina, 2018 vintage. Eight different red varieties were chosen: Gamay, Vranac, Pinot Noir, Zacinak, Black Tamjanika, Prokupac, Frankovka, and Shiraz. After collecting berries, seeds were manually removed, washed, and immediately air dried (moisture contents were below 10%). The seeds were then ground for precisely 15 s into a powder form (BOSCH domestic coffee mill), defatted (chloroform percolation for six hours at 70 °C) and used for preparing grape seed extracts (GSEs).

### 2.4. Experimental Design—Determination of Optimal Extraction Conditions

Box–Behnken experimental design in association with response surface methodology (RSM) was employed in order to set the optimal conditions to maximize the extraction yield of total phenols from grape seeds. We investigated the effects of three independent variables: x1—sample:solvent ratio (1:10 *w*/*v*, 2:10 *w*/*v*, 3:10 *w*/*v*); x2—time (20 min, 30 min, 40 min); and x3—temperature (25 °C, 50 °C, 75 °C) were investigated. In total, 16 experiments with 4 central points were carried out. Total phenolic content as determined by the Folin–Ciocalteu method was used as an output parameter. The experiment plan and experimentally obtained results are given in Table 1.

### 2.5. Extraction Procedure

Extraction was performed in an ultrasound bath (FALC, Treviglio, Italy) under the selected conditions (sample solvent ratio 1:10 *w*/*v*; 30 min; 50 °C) using two different extraction solvents—EtOH and ChCit. Thereafter, extracts were centrifuged (Janetzki T32 C, Wallhausen, Germany) for 15 min at 6000 rpm, and supernatants were decanted for further analysis.

### 2.6. HPLC–MS/MS Analyses of Phenolic Compounds in GSEs

The quantitative determination of phenolic compounds in GSEs was carried out on a Thermo Scientific chromatographic system consisting of an Accela quaternary pump, an autosampler and a PDA detector. Separations were performed on a Pursuit 3 PFP 150 × 4.6 mm, 3 µm (Agilent Technologies, Amstelveen, The Netherlands) at a flow rate of 800 µL min^−1^. The mobile phase was composed of solvent A (0.1% formic acid in water) and solvent B (acetonitrile) with gradient system elution of 0 min/5%, 22 min/35%, 24 min/100% and 25 min/5% followed by 5 min of column equilibration with 5% solvent B. The injection volume of the extracts was 5 μL (both EtOH and ChCit extracts were diluted prior the injection, 10 and 5 times, respectively). PDA detection was carried in the UV range from 200 nm to 400 nm. Following HPLC separation, eluent was introduced to the heated electrospray ionization (HESI) source of a TSQ Quantum Access MAX triple quadrupole mass analyzer (Thermo Fisher Scientific Inc., San Jose, CA, USA). Mass spectrometric analysis was performed in negative ionization mode with the optimum conditions as follows: the spray voltage was 2500 V with a tube lens offset of 50 V and skimmer offset of 0 V. The vaporizer temperature was set to 400 °C, while the capillary temperature was set to 300 °C. Nitrogen was used as the sheath gas (50 units) and auxiliary gas (10 units). TSQ Tune Software (Thermo Electron Corporation, Hemel Hepstead, UK) was used for automatic optimization of tuning parameters. Data acquisition was performed using Xcalibur 1.3 software (Thermo Electron Corporation, Hemel Hepstead, UK) for MS detection using selective reaction monitoring (SRM). Helium was used as a collision gas at a pressure of 1.5 mTorr. Table S1 (Supplementary Files) shows the molecular formulas, names and retention times of analytes and the SRM parameters, including *m*/*z* values of molecular and product ions, and collision energy. Peak integration and calibrations were performed using LC Quan™ software (Version 2.5.6, Thermo Electron Corporation, Hemel Hempstead, UK).

Analysis of phenolic compounds in GSEs was conducted as described in a recent study with some modification [26]. After establishing the analytical conditions that ensured good quantification of phenolic compounds in GSE samples, a protocol for method validation, in accordance with the recommendations of ICH Guideline Q2 (R1), included the following characteristics: linearity, limit of detection (LOD), limit of quantification (LOQ), precision and accuracy [28].

### 2.7. Total Phenol, Flavonoid and Flavan-3-ol Determination

Total phenolic content was determined using a rapid microassay as previously described [29]. Serial standard solutions of gallic acid and 200-fold diluted extracts (10 µL) were loaded on a 96-well microplate. Repeated volumes of 10-times diluted commercial Folin–Ciocalteu reagent (100 µL) and 1 M Na_2_CO_3_ (80 µL) were transferred to wells. After 60 min of incubation at room temperature in the dark, the absorbance was measured at 630 nm against a blank sample on a microtiter plate (MTP) reader (BIOTEK, Santa Clara, CA, USA, ELx800 Absorbance Microplate Reader). The results were expressed as mg gallic acid equivalents (GAE)/g of dry weight (mg GAE g^−1^ DW).

Quantitative analysis of total flavonoids was performed by a colorimetric assay [30]. Briefly, 10 μL of 100-fold diluted samples and epicatechin standards were added to 200 μL of distilled water and 30 μL of 5% NaNO_2_ solution in wells of MTP in triplicate. After 5 min at 37 °C, 30 μL of 10% AlCl_3_ solution was added, and the mixture was shaken. After 6 min, 20 μL of 1 M NaOH solution was added to the mixture. The reaction mixture was stirred, and absorbance compared to the blank solution was measured on an MTP reader at 490 nm. The results were expressed as mg of epicatechin equivalents (EE)/g of dry weight (mg EE g^−1^ DW).

Total flavan-3-ol content was estimated using a p-dimethylaminocinnamaldehyde (DMAC) microassay [31]. The temperature of the plate reader was set to 25 °C and allowed to equilibrate for at least 15 min prior to use. Standard solutions and 1000-fold diluted extracts (50 μL) were pipetted into wells of MTP in triplicate. Immediately after adding DMAC solution (250 μL) into all analysis wells, the plate was read against a reagent blank (ethanol) at 630 nm. The concentration of total flavan-3-ols was calculated from a calibration curve using B1 procyanidin as the standard. The results were expressed as mg of procyanidin B1 equivalents/g of dry weight (mg PB1E g^−1^ DW).

### 2.8. Antioxidant Activity

#### 2.8.1. Diphenylpicrylhydrazyl (DPPH) Radical Scavenging Microassay

DPPH radical scavenging ability was estimated as has been described with some modifications [32]. Diluted samples (100 times) and standard solutions (7 µL) were mixed with 193 µL of the DPPH radical solution (1.86 × 10^−4^ mol L^−1^ DPPH in ethanol, prepared ex tempore) in MTP wells. After incubation (1 h at room temperature), absorbance readings were taken at 490 nm on an MTP reader. Trolox was used as a standard for obtaining the calibration curve. Results were expressed as mM Trolox equivalents (TE)/g of dry weight (mM TE g^−1^ DW).

#### 2.8.2. FRAP (Ferric Ion Reducing Antioxidant Power) Microassay

FRAP assay was done as has been previously described with some modifications [33]. Briefly, FRAP working solution was prepared by mixing 300 mM acetate buffer (pH = 3.6), 10 mM TPTZ solution (i.e., 2, 4, 6-tripyridyl-s-triazine in 40 mM HCl) and 20 mM FeCl_3_ × 6H_2_O at a volume ratio of 10:1:1 and warmed at 37 °C for 10 min prior to use. Aliquoted samples (200-fold dilution) and Trolox solutions (20 µL) were added together with the FRAP working solution (280 µL) in 96-well microplates. Reaction mixtures were shaken and incubated at 37 °C for 30 min in dark conditions. Readings of the colored product (ferrous tripyridyltriazine complex) were taken at 630 nm using an MTP reader. Results were expressed as mM Trolox equivalents (TE)/g of dry weight (mM TE g^−1^ DW).

#### 2.8.3. ABTS/TEAC (Trolox Equivalent Antioxidant Capacity) Radical Scavenging Microassay

A TEAC test was performed as according to Pastoriza et al. [34]. Stock solutions of ABTS (7 mM) and potassium peroxodisulfate (2.45 mM) in phosphate buffer (pH = 7.4) were prepared and mixed in equal volumes. The mixture was left overnight to allow free-radical generation. After approximately 12–16 h, stock solution was diluted with phosphate buffer in order to achieve an absorbance of 0.7 ± 0.02 at 734 nm (1:80, *v*/*v*). Briefly, 20 μL of 1000-fold diluted extracts and Trolox solutions were mixed with 280 μL of the ABTS radical solution in 96-well microplates. After exactly 6 min, the absorbance readings were taken at 630 nm using an MTP reader. Radical scavenging ability was quantified using a Trolox calibration curve. Results were expressed as mM Trolox equivalents (TE)/g of dry weight (mM TE g^−1^ DW).

#### 2.8.4. CUPRAC (Cupric Ion Reducing Antioxidant Capacity) Microassay

CUPRAC assay was performed as according to Zengin et al. [35]. Aliquoted extracts (1000-times diluted) and Trolox solutions (67 µL) were pipetted into 96-well microplates. After that, 61 µL of 0.01 M CuCl_2_, 61 µL of 7.5 × 103 M neocuproine in ethanol and 61 µL of ammonium acetate buffer (pH = 7) were added. After 30 min of incubation, absorbance readings were made against a reagent blank at 450 nm. Results were expressed as mM Trolox equivalents (TE)/g of dry weight (mM TE g^−1^ DW).

### 2.9. Statistical Analysis

Statistical analysis was performed using software programs SPSS 20 (SPSS Inc., Chicago, IL, USA) and GraphPad Prism 6. The results were expressed as mean values with corresponding standard deviations. Prior to statistical processing, the data were tested for homogeneity and normal distribution. Extraction efficiency of applied solvents was compared by Student’s t-test. Pearson’s correlation coefficient was used to check the relationship between the variables. Differences between varieties were observed by Bonferroni correction using software Past 3.25. In all statistical tests, values of *p* < 0.05 were considered significant. Response surface methodology was performed using Design Expert Statistical Software package 7.0.0. (Stat Ease, Inc., Minneapolis, MN, USA). Analysis of variance (ANOVA) was used to test the adequacy of the mathematical model derived from the experimental data.

## 3. Results and Discussion

### 3.1. Experimental Design—Determination of Optimal Extraction Conditions

Box–Behnken design was applied to select optimal phenol extraction conditions, and within it, a quadratic model proved to be the most suitable. The coefficient b1^2^ was not statistically significant (*p* < 0.05) and was excluded from the model. The adequacy of the obtained model was confirmed by lack-of-fit test, R^2^, adjusted R^2^ (adj. R^2^) and predicted R^2^ (pred. R^2^). The adj. R^2^ = 0.9494, pred. R^2^ = 0.8203 and the lack-of-fit test (*p* > 0.05) confirmed good accuracy of the derived model. Moreover, the coefficient of variance of 3.81 clearly stated the consistency of the observed values. The final mathematical model as determined from the experimental results in terms of coded factors is given as Equation (1):

TPC = 86.87 − 14.16 × x1 + 0.58 × x2 + 1.08 × x3 − 3.69 × x1 × x2 + 0.71 × x1 × x3 + 2.87 × x2 × x3 + 4.74 × x2^2^ − 15.71 × x3^2^ (1) (1)

Coefficients corresponding to x1, x1x2, x2^2^ and x3^2^ are significant model terms. The sample:solvent ratio (x1) had the highest impact on phenol extraction yield (*p* < 0.0001). Interaction x1x2 proved to be significant, so the mutual influence of sample:solvent ratio and extraction time has to be carefully balanced. Highly significant quadratic terms for x1^2^ and x2^2^ indicated the effect of sample:solvent ratio and extraction time were not linear. Extraction time (x2) was the factor with the lowest impact on extraction efficiency, so the central point of this factor (30 min) was chosen for further optimization. Response surface plots (Figure S1A,B) showed that within 30 min, maximal extraction can be reached with the lowest sample:solvent ratio and at a temperature of 50 °C. Namely, TPC yield slightly decreased when the temperature changed from 50 °C to 75 °C. This could be explained by phenol compounds’ vulnerability to high temperatures. Therefore, the optimal conditions for obtaining extracts with the highest total phenolic content are suggested: sample:solvent ratio 1:10 *w*/*v*, extraction time 30 min and extraction temperature 50 °C. Following the defined parameters, we aimed to investigate phenol composition, total phenolic content, total flavonoid content, total flavan-3-ols and antioxidant activity of seed extracts (both EtOH and ChCit) derived from eight different grape varieties.

### 3.2. Validation of HPLC–MS/MS Method for Phenolic Compounds Assay

The modified HPLC–MS/MS method has been fully validated and has been found to be suitable for GSE analysis in a total time of 30 min. Figure S2 (Supplementary Files) shows very good separation of the 10 compounds in standard solution on the Pursuit 3 PFP 150 × 4.6 mm HPLC column.

Mass fragmentation patterns of all analytes (Supplementary Files—Figure S2 and Table S1) were consistent with literature reports. Mallic acid with a parent [M-H]^−^ ion at *m/z* 133 typically produced product ion *m*/*z* 115 corresponding to loss of H_2_O. A product ion of tartaric acid at *m*/*z* 87 was yielded by combinations of losses COO and H_2_O [M-H-62]^−^. The hydroxybenzoic acids (gallic and protocatechuic acid) produced same transitions, resulting from loss of COO, with *m*/*z* 169→125 and 153→109, respectively. Catechin and epicatechin showed [M-H]^−^ molecular ion peaks at *m*/*z* 289 that produced the same fragmentation pattern distinguished at *m*/*z* 245, which corresponded to the loss of a (CH)_2_OH group [M-H-44]^−^. Two peaks with molecular ion [M-H]^−^ at *m*/*z* 305, characteristic for gallocatechin and epigallocatechin, showed the fragmentation pattern corresponding to the neutral loss of a tryhydroxybenzene ring and product ions at *m*/*z* 125. Compounds with [M-H]^−^ ions at *m*/*z* 577 were assigned to procyanidin dimers (B1 and B2) with daughter ions at *m*/*z* 289, corresponding to interflavan bond cleavage [M-H-288]^−^.

Calibration curves were linear over a wide range of concentrations, and the least-calculated R^2^ was 0.9920 (Supplementary Files—Table S2). LOD and LOQ values are also shown in Table S2.

HPLC–MS/MS was found to have good accuracy and repeatability (Supplementary Files—Table S3). Accuracy varied between 63.60% and 119.34%, while precision had RSD values lower than 20%. Unsatisfactory results were only obtained in the analysis of tartaric acid in the ChCit GSEs. The response of tartaric ions in the mass analyzer was most likely suppressed due to the predominant presence of citric ions in ChCit GSEs as well as high similarity with citric ions in the solvent.

### 3.3. HPLC–MS/MS Determination of Phenolic Compounds in GSEs

HPLC was applied to assess the effect of grape variety on phenolic composition of red grape seeds. In addition, phenol derivation was performed using EtOH (conventional solvent) and ChCit (NADES) in order to evaluate the impact of extraction solvent. The investigation was done on eight different grape varieties, and the obtained results indicate evident differences between samples, which is in agreement with already published data [36]. Observed distinctions come from genotypic and phenotypic variability of grapes. In addition, different factors can affect the phenol composition, such as climatic conditions, seasonal variations of weather, soil conditions, agronomical practices, etc. [37,38]. Our previous research was conducted on grapes harvested in the 2017 season, and some samples were of the same varieties [39]. Comparing the results obtained for the two consecutive years, we observed significant differences within the same varieties, confirming the influence of multiple factors. As expected, extraction solvent also had a notable impact on the composition of phenolic compounds.

Phenolic composition is shown in Table 2 (EtOH GSEs) and Table 3 (ChCit GSEs). Among quantified acids in ethanol extracts, tartaric acid was the most abundant in almost all varieties, followed by malic acid. In contrast, the ChCit solvent mainly enhanced malic acid extraction (except in Pinot Noir and Black Tamjanika, as they were characterized by the highest gallic acid content). Seeds from Prokupac had significantly higher gallic acid content in comparison with all other varieties—a previously reported finding [36]. Protocatechuic acid was present in minor quantities across all samples.

With respect to the most abundant phenols in GSEs, monomer flavan-3-ols (catechins), four different compounds were found. Regardless of the extraction solvent, (+)-catechin and (−)-epicatechin were the main structures quantified in all samples. In previously published data for grape seed extracts, similar patterns were observed. Namely, some authors have reported that (+)-catechin was more abundant than (−)-epicatechin [17,40]; meanwhile, others noticed the domination of (−)-epicatechin [41,42]. In our samples, catechin was present in higher quantities than epicatechin in almost all varieties; (−)-epicatechin was dominant only in Zacinak and Gamay ethanol seed extracts and in Frankovka seed extracts obtained using both solvents. Within varieties, Pinot Noir had the highest amount of (+)-catechin (6.978 and 3.126 mg/g of dry weight in ethanol and ChCit GSEs, respectively) and (−)-epicatechin (4.782 and 1.950 mg/g of dry weight in ethanol and ChCit GSEs, respectively). Based on variety, obtained results were in accordance with recent findings (Pinot Noir had significantly higher catechin content than Shiraz) [34]. Catechin gallic derivatives were present in trace amount or not detected. In addition, dimer phenols (procyanidin B1 and B2) were ascribed to GSEs. Isomer B1 was measured at much higher concentrations independent of extraction medium or grape variety. Such a finding correlates with previously published data [43,44]. Both cited studies identified B1 as most prevalent in the Shiraz procyanidin profile.

Overall, ethanol showed better extraction efficiency than ChCit (sum of quantified phenols by HPLC was much higher 70.53 vs. 40.47 mg/g DW). Nevertheless, different phenol classes were extracted selectively by the conventional and the green solvent. Data shown in Table 2 and Table 3 demonstrate that ChCit showed notably higher affinity for extracting procyanidins from particular grape varieties. This finding is in accordance with previously reported observations that acid-based NADES possess excellent extraction performance for flavan-3-ols [45]. Finally, regardless of the similar polarity of ChCit and EtOH, their chemical structures cause differences in other physicochemical characteristics such as viscosity, density and pH, and thus defines extraction efficiency toward specific compounds [46]. Modeling compound/solvent interactions represents a future topic for extraction research [47]. Therefore, solvents such as NADES, with designable properties, should completely fulfill this demand.

### 3.4. Content of Total Phenolics, Flavonoids and Flavan-3-ols

**Figure 1:**
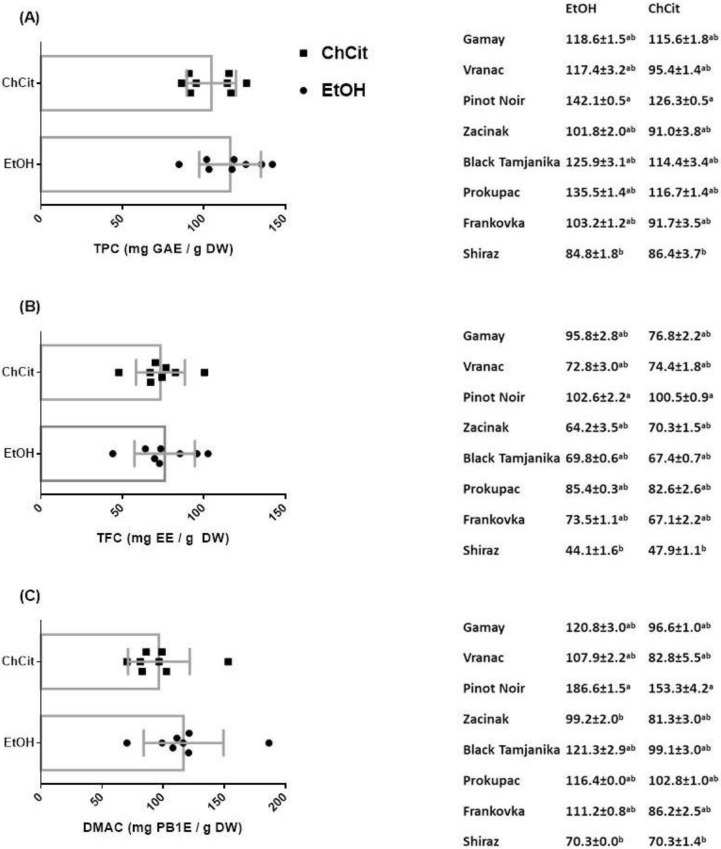
Total phenolic content (**A**); total flavonoid content (**B**) and total flavan-3-ol content (**C**) of GSEs. Data are expressed as mean (*n* = 8) ± SD for solvent impact and as mean (*n* = 3) ± SD for variety impact. Lower-case letters indicate significant difference in each group (*p* < 0.05) as measured by Bonferroni test. Legend: GSEs—grape seed extracts; ChCit—choline chloride:citric acid; EtOH—ethanol; DW—dry weight; TPC—total phenolic content; GAE—gallic acid equivalents; TFC—total flavonoid content; EE—epicatechin equivalents; DMAC—4-dimethylaminocinnamaldehyde; PB1E—procyanidin B1 equivalents.

Total phenolic content of extracts fluctuated remarkably. Such data distribution can be explained by different factors such as: climate conditions and seasonal variations, soil characteristics, varietal characteristics, environmental conditions, agronomical practices, sample management, and so on [37,38,48]. Herein, some of the factors can be excluded. Namely, all grape samples were obtained during the same year and at technological maturity. Sample preparation and extraction were uniform. We can assume that the differences revealed in this study primarily come from variety characteristics. The highest TPC values were found in Pinot Noir seed extracts obtained using both solvents (142.1 ± 0.5 and 126.3 ± 0.5 mg GAE/g DW for ethanol and ChCit extracts, respectively). Obtained results were in agreement with previous investigations that found Pinot Noir to have high polyphenol content [36,49]. Namely, total phenolic content in Pinot Noir grape seed extracts was found by Pantelic et al. to be 102.98 mg GAE/g DW, while Radulescu et al. reported TPC values of 169.53 and 77.05 mg GAE/g DW from an organic and conventional vineyard, respectively. Both previously published results were in line with TPC values obtained in this study. The lowest TPC was observed in seeds of Shiraz (84.8 ± 1.8 and 86.4 ± 3.7 mg GAE/g DW for ethanol and ChCit extracts, respectively). Pantelic with her team analyzed phenolic content of the same three varieties (Prokupac, Pinot Noir and Shiraz) [34]. Interestingly, they also reported that Pinot Noir had a significantly higher TPC content in comparison with Shiraz (right side of Figure 1), which supports our assumption. Results obtained for Vranac seeds were compared with previously published data on the same variety, and our values were slightly higher, which can be related to seasonal variability [50]. In terms of TPC, there was no significant difference between conventional and green solvent, although ethanol displayed slightly higher efficiency. TPC was strongly correlated with both TFC and total flavan-3-ol content (r = 0.841 and 0.905 for ethanol, and r = 0.881 and 0.952 for ChCit extracts, respectively; *p* < 0.01). A very similar value distribution was obtained for total flavonoid content, with both solvents yielding around 75 mg EE/g DW on average. In particular, TFC varied between 44.1 and 102.6 EE/g DW for EtOH and 47.9 and 100.5 mg EE/g DW for ChCit extracts. Results obtained for total flavan-3-ol content, as the phenol group most-specific to grape seeds, also differed from variety to variety. As reported before, flavan-3-ol diversity in grape seeds is primarily influenced by species, variety and environment [51]. Finally, even though higher values were observed for EtOH extracts, statistical significance was not reached (*p* = 0.1892).

Diluted ethanol is known to be suitable for ultrasound-assisted phenol extraction from grape samples [52]. Taking into account solvent influence on extraction of all three phenol classes (Figure 1), the high efficiency of ethanol is confirmed. As a matter of fact, though higher values were obtained for total phenolic, flavonoid and flavan-3-ol contents in the ethanol group (significant difference was absent: *p* = 0.2013, *p* = 0.7568 and *p* = 0.1892, respectively), lower standard deviations were noticed when ChCit was applied, especially for the flavan-3-ol group. High affinity of structurally different NADES towards flavan-3-ols has been observed before [53]. Alrugaibah et al. pointed out that NADES consisting of choline chloride:proline:malic acid 1:1:1 and 30% water is the most promising one for catechin extraction [21]. Therefore, ChCit could be featured as a green solvent with good potential for homogeneous phenol extraction from grape seeds and other food waste materials. Such a phenomenon should be extended from the laboratory to industry due to its dual environmentally positive effects of applying eco-friendly extraction to effectively draw out the vast majority of bioactives from food waste material.

### 3.5. Antioxidant Activity of GSEs

**Figure 2:**
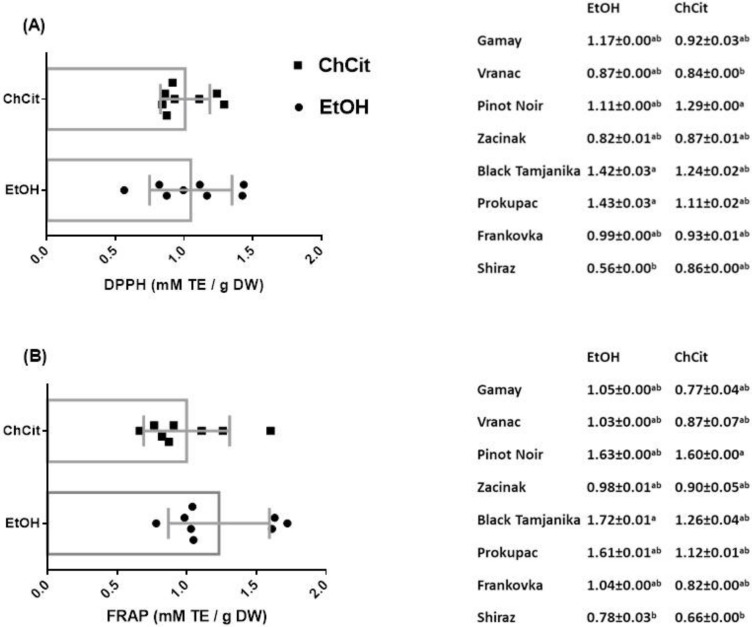

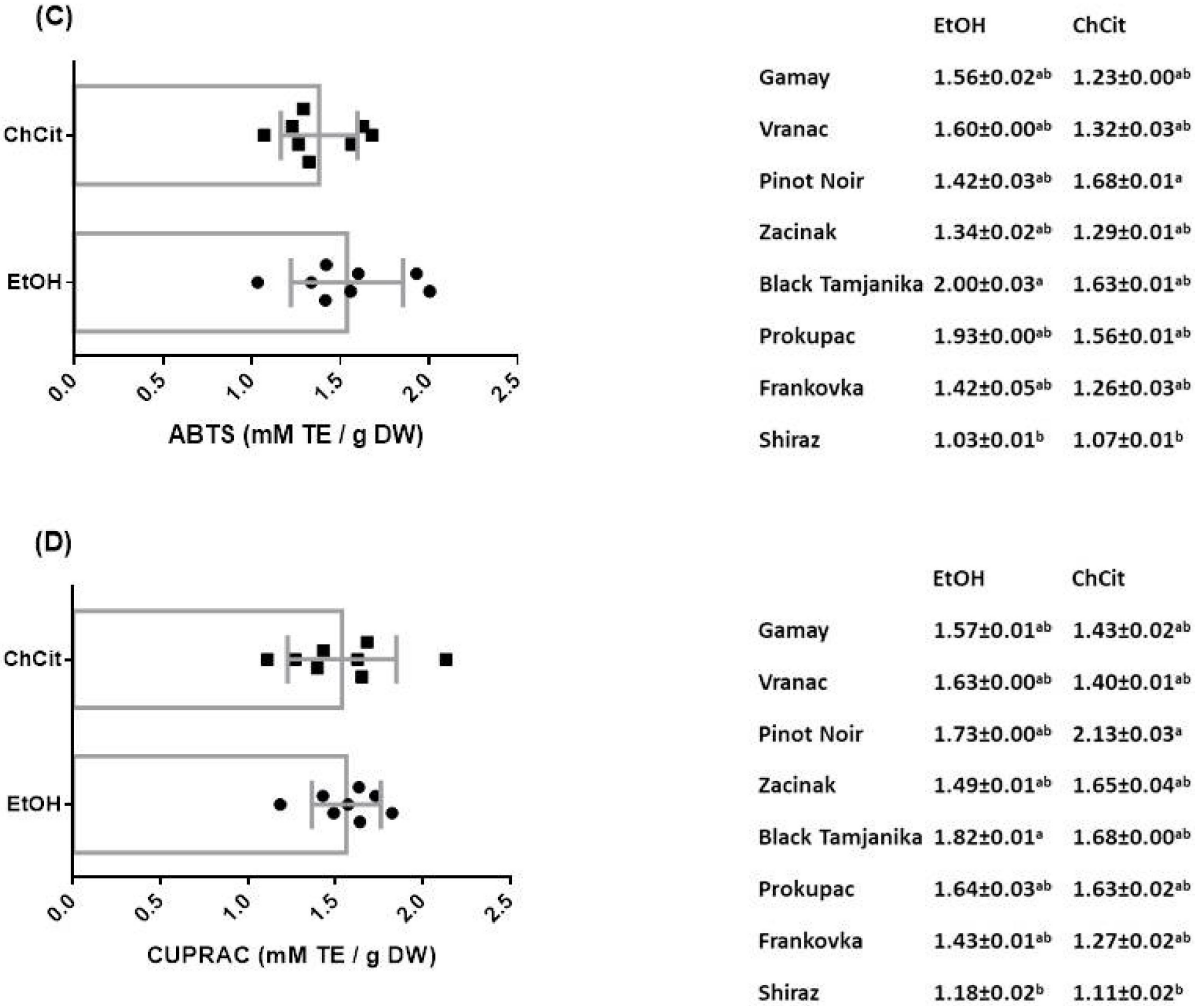
Antioxidant activity: DPPH—diphenylpicrylhydrazyl (**A**); FRAP—ferric ion reducing antioxidant power (**B**); ABTS—2,2′-azino-bis-3-ethylbenzothiazoline-6-sulfonic acid (**C**); and CUPRAC—cupric ion reducing antioxidant capacity (**D**) of GSEs. The data are expressed as mean (*n* = 8) ± SD for solvent impact and as mean (*n* = 3) ± SD for variety impact. Lower-case letters indicate significant difference in each group (*p* < 0.05) as measured by Bonferroni test. Legend: GSEs—grape seed extracts; ChCit—choline chloride:citric acid; EtOH—ethanol; DW—dry weight; TE—Trolox equivalents.

Phenols have an important protective role against oxidative stress-mediated pathological processes. In short, these compounds exhibit their antioxidant activity through different mechanisms. Therefore, in an attempt to include all the possible reaction mechanisms of antioxidants, many in vitro methods have been suggested and updated. Each of them has its advantages, disadvantages and limitations. As individual tests cover different segments of antioxidant action, it is always good to apply several of them on the same material. Therefore, we decided to assess the antioxidant activity of GSEs by measuring the reduction of stable DPPH and ABTS radicals in the presence of phenolic compounds. On the other hand, FRAP and CUPRAC tests were selected due to their similar method of action: they measure the ability of antioxidants to reduce a metal ion (Fe^3+^ and Cu^2+^, respectively) in specific conditions [54]. Obtained results are showed in Figure 2.

The left side of Figure 2 represents how solvent influenced GSE antioxidant activity; it can be observed that ethanol and ChCit did not show a significant difference in DPPH (*p* = 0.7473), FRAP (*p* = 0.1906), ABTS (*p* = 0.2678) and CUPRAC (*p*= 0.8486) tests. However, it is good to point out that DPPH and ABTS seem promising based on the modest standard deviations in these tests, particularly when ChCit was applied as a solvent. Additionally, the results of the CUPRAC test are almost the same regardless of the used solvent, so it could a good option for measuring the antioxidant activity of green extracts derived from food waste. Within varieties (right side of Figure 2), antioxidant activity ranged from 0.56 to 2.00 mM TE g^−1^ DW of GSEs, with a special emphasis on Black Tamjanika, taking into account results from all four tests and both solvents. Thus, this variety could be a source of other antioxidant compounds besides phenolics since it did not highly stand out regarding total phenol, flavonoid and flavan-3-ol contents (right side of Figure 1). It is an old Serbian variety, characteristic of the eastern part of the country, and to the best of our knowledge, this variety has not been explored in the past. One very recent study found Tamjanika seeds as a good source of carotenoids and tocopherols [55]. Given that they are known antioxidant substances, this can be a potential explanation for our result regarding the biopotential of Black Tamjanika—autochthonous variety.

Correlations between antioxidant activity and different classes of phenolics have been evaluated through numerous studies. Xu et al. reported very high correlation between antioxidant activity (three tests were performed) and total phenolics, total flavonoids and total flavan-3-ols [56]. Further, our research group revealed a similar pattern based on investigation of varieties collected the year before [39]. Although the number of studies related to NADES increases every year, there is still a vigorous need for their deeper exploitation. Two recent up-to-date studies figured out the possibility of using NADES as a solvent for obtaining high-value extracts with strong antioxidant activity [21,57].

**Figure 3:**
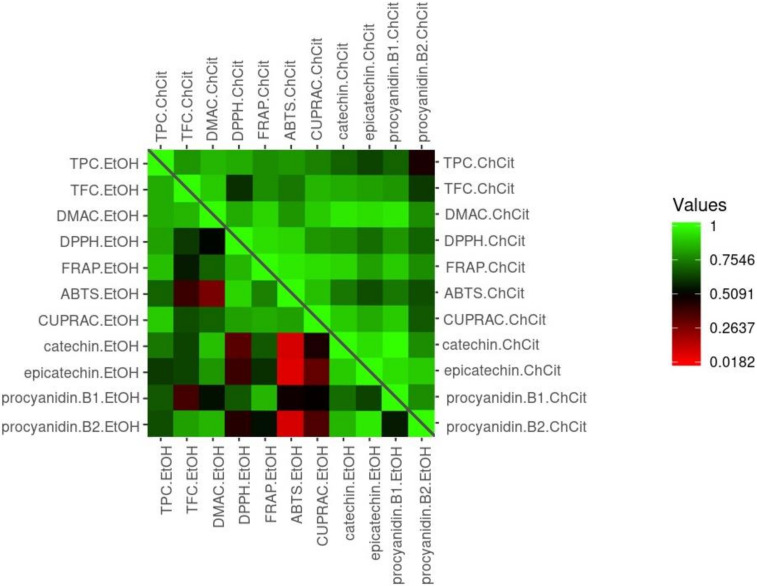
Heatmap of the correlations between antioxidant activity and phenolic compounds. Legend: ChCit—choline chloride:citric acid; EtOH—ethanol; TPC—total phenolic content; TFC—total flavonoid content; DMAC—4-dimethylaminocinnamaldehyde; DPPH—diphenylpicrylhydrazyl; FRAP—ferric ion reducing antioxidant power; ABTS—2,2′-azino-bis-3-ethylbenzothiazoline-6-sulfonic acid; CUPRAC—cupric ion reducing antioxidant capacity.

Correlations between antioxidant activity and total phenols, total flavonoids, total flavan-3-ols, catechins and procyanidins can be visualized using a heatmap (Figure 3). Observing total contents of different phenolic compounds in EtOH extracts of grape seed, significant correlations were pointed out in only three cases: TPC and DPPH test (r = 0.808), TPC and FRAP test (r = 0.874), and TPC and CUPRAC (r = 0.884). Apart from these, correlations between different phenolic compounds and antioxidant activity for ChCit extracts were all significant except for one (TFC and DPPH test). A recently published study also revealed a strong correlation between antioxidant potential, measured by DPPH, FRAP and ABTS assays, and TPC in *Lavandula* after NADES extraction [58]. For a general sense of the present investigation, Pearson’s correlation coefficients ranged from 0.092 ((−)-epicatechin content and antioxidant activity by ABTS in EtOH extracts) to 0.907 ((+)-catechin content and antioxidant activity by FRAP in ChCit extracts). The observed relationship between the data for the green approach (right diagonal half of the square) demonstrates that ChCit is a more-suitable medium for GSE antioxidant activity preservation. Therefore, ChCit GSEs could potentially be utilized as a powerful raw material for dietary supplements designed to prevent compromised redox balance.

## 4. Conclusions

Grape seeds carry abundant amounts of biologically active phenol compounds, and this study quantified a select set of those present in eight grape varieties. The samples were characterized predominantly using flavan-3-ols ((+)-catechin and (−)-epicatechin), followed by procyanidins dimers. Phenolic acids were also present, but in small amounts. The mentioned compounds are responsible for the strong antioxidant properties of grape seeds (ranging from 0.56 to 2.00 mM TE g^−1^ DW of GSEs based on DPPH, FRAP, ABTS and CUPRAC tests). Basically, the aim of the present study was to optimize the extraction process of phenols by investigating three important factors. Optimal conditions for obtaining extracts with the highest total phenolic content were suggested: sample:solvent ratio 1:10 *w*/*v*, extraction time 30 min and extraction temperature 50 °C. Furthermore, the impact of the extraction solvent was evaluated. Although a conventional solvent (ethanol) showed slightly higher efficiency in extracting phenolic compounds, intriguing results concerning a green solvent (ChCit) were obtained. Namely, (1) ChCit had a strong affinity toward procyanidins; (2) ChCit displayed lower standard deviations in spectrophotometric assays; and finally, (3) correlation between antioxidant activity and quantified phenolic compounds was stronger in the case of the green solvent. Keeping in mind the necessity of protecting the environment, we suppose that the use of NADES in extracting valuable compounds from food waste material has a bright future. It is important to emphasize that, despite many advantages, such as stability, low costs and green properties, the usage of NADES has some drawbacks, primarily high viscosity. More detailed exploration of green extracts is needed; nevertheless, the present study confirms and enhances NADES application in pharmaceutical, cosmetic and food industries.

## Figures and Tables

**Table 1:** Actual values of the independent variables in BBD and experimental results.

Run Order	x1 Sample:Solvent Ratio (*w*/*v*)	x2 Time (min)	x3 Temperature (°C)	Y—Experimental TPC (mg GAE/g DW)
1	1:10	30	75	86.24
2	2:10	30	50	84.22
3	2:10	20	25	79.85
4	3:10	20	50	78.89
5	2:10	40	25	70.97
6	2:10	30	50	89.70
7	3:10	40	50	76.95
8	2:10	30	50	86.41
9	1:10	40	50	110.75
10	3:10	30	25	53.69
11	1:10	20	50	97.92
12	2:10	20	75	76.08
13	2:10	30	50	89.13
14	3:10	30	75	57.44
15	1:10	30	25	85.34
16	2:10	40	75	78.69

Legend: BBD—Box–Behnken design; TPC—total phenolic content; GAE—gallic acid equivalents; DW—dry weight.

**Table 2:** Phenolic compounds quantified in EtOH GSEs (results are expressed as mg/g of dry weight).

	Variety							
	Gamay	Vranac	Pinot Noir	Zacinak	Black Tamjanika	Prokupac	Frankovka	Shiraz
Phenolic acids								
TA	1.279 ^bc^	1.413 ^abc^	1.719 ^abc^	1.228 ^c^	2.844 ^a^	1.690 ^abc^	2.501 ^ab^	1.501 ^abc^
MA	1.088 ^abc^	0.901 ^abc^	1.313 ^abc^	0.362 ^c^	1.526 ^ab^	1.853 ^a^	0.640 ^bc^	1.327 ^abc^
GA	0.172 ^c^	0.308 ^abc^	0.365 ^abc^	0.580 ^ab^	0.470 ^abc^	0.911 ^a^	0.275 ^bc^	0.318 ^abc^
PA	0.011 ^ac^	0.015 ^ac^	0.008 ^c^	0.019 ^ac^	0.008 ^c^	0.020 ^ac^	<LOQ	0.027 ^a^
Catechins								
GC	0.004 ^ab^	nd	0.004 ^ab^	0.003 ^b^	0.004 ^ab^	0.005 ^a^	0.003 ^b^	nd
EGC	0.012 ^ab^	nd	nd	0.010 ^ab^	0.018 ^ab^	0.022 ^ab^	0.026 ^a^	0.005 ^b^
C	1.256 ^abc^	1.309 ^abc^	6.978 ^a^	1.238 ^c^	2.000 ^abc^	3.366 ^ab^	1.709 ^abc^	1.250 ^bc^
EC	1.393 ^abc^	0.623 ^c^	4.782 ^a^	1.406 ^abc^	1.595 ^abc^	3.107 ^ab^	2.950 ^abc^	1.080 ^bc^
Procyanidins								
PB1	1.444 ^abc^	1.134 ^bc^	2.267 ^ab^	1.083 ^c^	2.162 ^abc^	2.269 ^a^	1.337 ^abc^	1.617 ^abc^
PB2	0.054 ^ab^	0.012 ^b^	0.112 ^a^	0.018 ^ab^	0.026 ^ab^	0.065 ^ab^	0.065 ^ab^	0.013 ^b^

Values represent mean of three replicates. Standard deviation was <5%. Lower-case letters indicate significant difference in each group (*p* < 0.05) as measured by Bonferroni test. Legend: EtOH—ethanol; GSEs—grape seed extracts; TA—tartaric acid; MA—malic acid; GA—gallic acid; PA—protocatechuic acid; GC—gallocatechin; EGC—epigallocatechin; C—(+)-catechin; EC—(−)-epicatechin; PB1—procyanidin B1; PB2—procyanidin B2; LOQ—limit of quantification; nd—not detected.

**Table 3:** Phenolic compounds quantified in ChCit GSEs (results are expressed as mg/g of dry weight).

	Variety							
	Gamay	Vranac	Pinot Noir	Zacinak	Black Tamjanika	Prokupac	Frankovka	Shiraz
Phenolic acids								
TA	1.121 ^a^	0.349 ^abc^	0.129 ^c^	0.383 ^abc^	0.201 ^abc^	0.724 ^ab^	0.161 ^bc^	0.510 ^abc^
MA	1.028 ^ab^	0.806 ^ab^	0.175 ^b^	0.822 ^ab^	<LOQ	1.050 ^a^	0.635 ^ab^	0.888 ^ab^
GA	0.140 ^c^	0.240 ^abc^	0.294 ^abc^	0.516 ^a^	0.326 ^abc^	0.509 ^ab^	0.183 ^bc^	0.241 ^abc^
PA	0.007 ^bc^	0.017 ^ab^	0.008 ^abc^	0.010 ^abc^	0.014 ^abc^	0.009 ^abc^	0.006 ^c^	0.026 ^a^
Catechins								
GC	nd	nd	nd	nd	0.004 ^a^	0.004 ^a^	0.003 ^b^	nd
EGC	0.001 ^a^	0.001 ^a^	0.001 ^a^	0.002 ^a^	0.005 ^a^	0.002 ^a^	0.007 ^a^	0.001 ^a^
C	0.672 ^abc^	0.712 ^abc^	3.126 ^a^	0.911 ^abc^	1.018 ^abc^	1.220 ^ab^	0.619 ^bc^	0.469 ^c^
EC	0.494 ^abc^	0.184 ^bc^	1.950 ^a^	0.608 ^abc^	0.494 ^abc^	0.714 ^abc^	0.769 ^ab^	0.121 ^c^
Procyanidins								
PB1	1.350 ^abc^	1.247 ^c^	3.833 ^a^	1.611 ^abc^	1.743 ^abc^	2.026 ^ab^	1.307 ^bc^	1.311 ^abc^
PB2	0.027 ^bc^	0.031 ^abc^	0.080 ^a^	0.037 ^abc^	0.049 ^abc^	0.038 ^abc^	0.062 ^ab^	0.024 ^c^

Values represent mean of three replicates. Standard deviation was <5%. Lower-case letters indicate significant difference in each group (*p* < 0.05) as measured by Bonferroni test. Legend: ChCit—choline chloride: citric acid; GSEs—grape seed extracts; TA—tartaric acid; MA—malic acid; GA—gallic acid; PA—protocatechuic acid; GC—gallocatechin; EGC—epigallocatechin; C—(+)-catechin; EC—(−)-epicatechin; PB1—procyanidin B1; PB2—procyanidin B2; LOQ—limit of quantification; nd—not detected.

## Data Availability

Data are contained within the article.

## Supplementary Materials

The following supporting information can be downloaded at: https://www.mdpi.com/article/10.3390/antiox11081595/s1, Table S1: HPLC–MS/MS acquisition parameters used for the analysis of phenol compounds in grape seed extracts (GSEs); Table S2: Results for LOD, LOQ and linearity of HPLC–MS/MS method for phenolic compound assay in GSEs; Table S3: Results for accuracy (recovery) and precision (RSD) of HPLC–MS/MS; Figure S1: Contour plot (A) and three-dimensional (3D) response surface (B); Figure S2: HPLC–MS/MS chromatograms.
