# Supplementary material for: Optimization of Extraction and HPLC–MS/MS Profiling of Phenolic Compounds from Red Grape Seed Extracts Using Conventional and Deep Eutectic Solvents
Source: Antioxidants (Basel). 2022 Aug 18;11(8):1595. doi: 10.3390/antiox11081595 (PMC9405313; doi:10.3390/antiox11081595)
Supplement: Supplementary file 1 [file antioxidants-11-01595-s001.zip › Figure S2.pdf]

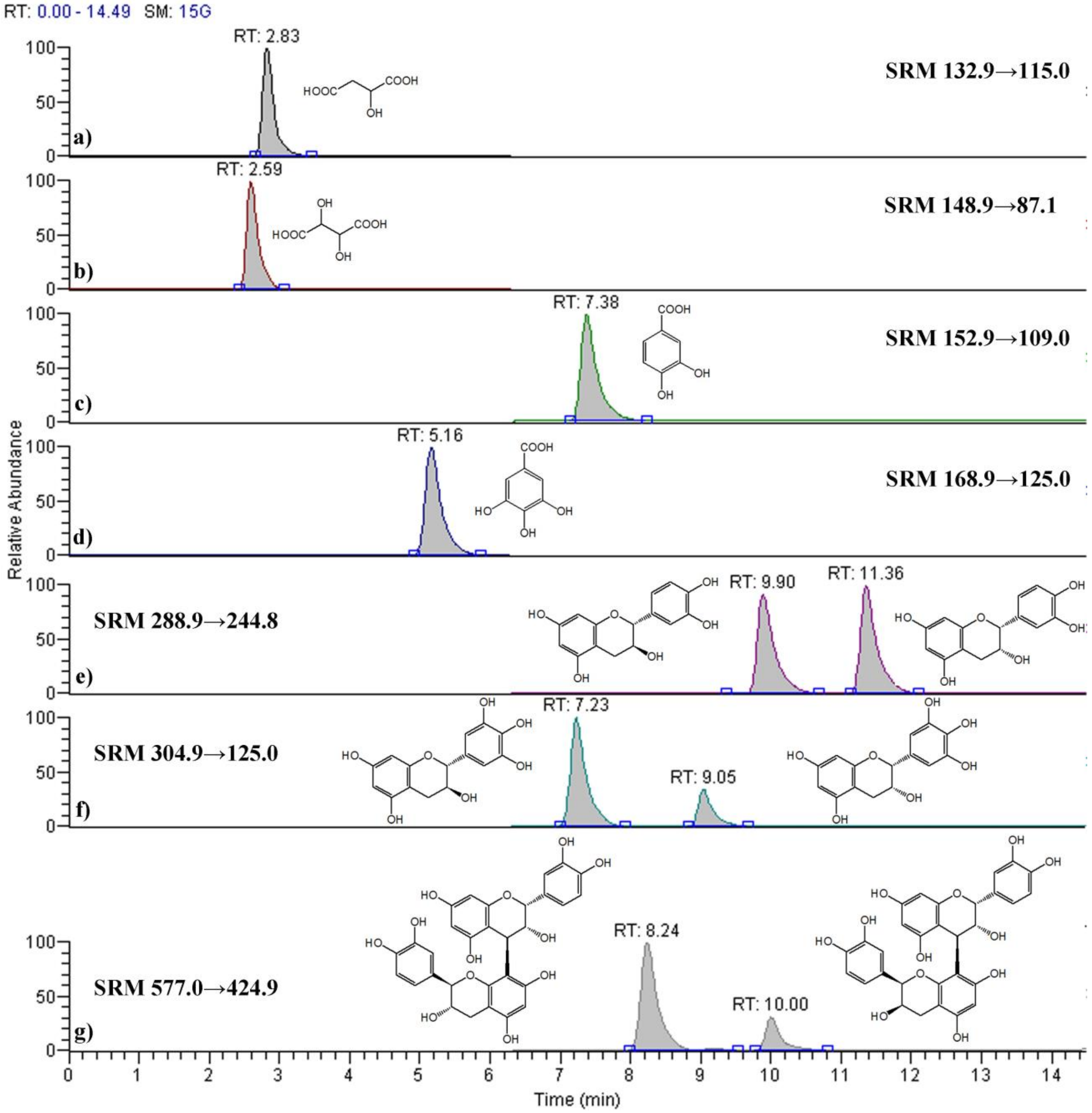

Figure S2. HPLC-MS/MS chromatograms

a) Malic acid; b) Tartaric acid; c) Protocatechuic acid; c) Gallic acid; d) (+)-Catechin and (-)-Epicatechin; e) Gallocatechin and Epigallocatechin; g) Procyanidin B1 and Procyanidin B2

Legend: RT – retention time; SRM - selective reaction monitoring
