# Supplementary material for: Optimization of Extraction and HPLC–MS/MS Profiling of Phenolic Compounds from Red Grape Seed Extracts Using Conventional and Deep Eutectic Solvents
Source: Antioxidants (Basel). 2022 Aug 18;11(8):1595. doi: 10.3390/antiox11081595 (PMC9405313; doi:10.3390/antiox11081595)
Supplement: Supplementary file 1 [file antioxidants-11-01595-s001.zip › Table S1.pdf]

Table S1. HPLC-MS/MS acquisition parameters used for the analysis of phenol compounds in Grape Seed Extracts (GSEs).

Compound	Abbrev.	Formula	Retention time (min)	$[M-H]^-$ Experimental (*m/z*)	MS/MS Fragment	Collision Energy (V)
**Tartaric acid**	TA	$C_4H_6O_6$	2.59	148.9	103.1	13
**Malic acid**	MA	$C_4H_6O_5$	2.83	132.9	115.0	14
**Gallic acid**	GA	$C_7H_6O_5$	5.16	168.9	125.0	16
**Gallocatechin**	GC	$C_{15}H_{14}O_7$	7.23	304.8	124.9	21
**Protocatechuic acid**	PA	$C_7H_6O_4$	7.38	152.9	109.0	14
**Procyanidin B1**	PB1	$C_{30}H_{26}O_{12}$	8.24	577.0	288.8	27
**Epigallocatechin**	EGC	$C_{15}H_{14}O_7$	9.05	304.9	125.0	25
**(+) Catechin**	C	$C_{15}H_{14}O_6$	9.90	288.9	244.8	18
**Procyanidin B2**	PB2	$C_{30}H_{26}O_{12}$	10.00	577.0	288.8	27
**(-) Epicatechin**	EC	$C_{15}H_{14}O_6$	11.36	288.9	244.9	17
