# Supplementary material for: Optimization of Extraction and HPLC–MS/MS Profiling of Phenolic Compounds from Red Grape Seed Extracts Using Conventional and Deep Eutectic Solvents
Source: Antioxidants (Basel). 2022 Aug 18;11(8):1595. doi: 10.3390/antiox11081595 (PMC9405313; doi:10.3390/antiox11081595)
Supplement: Supplementary file 1 [file antioxidants-11-01595-s001.zip › Table S2.pdf]

Table S2. Results for LOD, LOQ and linearity of HPLC-MS/MS method for phenolic compounds assay in GSEs

Compound	Concentration interval (mg/L)	Linear equation	Linearity R^2	LOD (mg/L)	LOQ (mg/L)
TA	0.6-60	y=28078x+48520	0.9945	0.20	0.6
MA	2-100	y=42868x+279878	0.992	0.08	0.25
GA	0.2-20	y=73157x+40746	0.9976	0.07	0.2
GC	0.2-10	y=19056x-5054.9	0.9949	0.07	0.2
PA	0.02-2	y=169349x+9223.2	0.9998	0.01	0.02
PB1	0.1-100	y=27233x+7839.3	0.9998	0.03	0.1
EGC	0.1-100	y=8962.7x-154.43	0.9969	0.03	0.1
C	2-200	y=22311x+79977	0.9994	0.17	0.5
PB2	0.16-160	y=3576.3x+5640.5	0.9981	0.05	0.16
EC	2-200	y=22734x+132115	0.9978	0.67	2

Legend: LOD- Limit of Detection; LOQ- Limit of Quantification; TA- Tartaric Acid; MA- Malic Acid; GA- Gallic Acid; GC- Gallocatechin; PA- Protocatechuic Acid: PB1- Procyanidin B1; EGC- Epigallocatechin; C- (+)-Catechin: PB2-Procyanidin B2; EC- (-)-Epicatechin

To determine the concentrations of phenolic compounds from the peak areas calibration curves (x-axis: standard concentrations; y-axis: peak areas) were constructed. Standards for the calibration curves points were obtained by the series of stock standard dilutions and included seven different concentrations. The linearity of the calibration curves was evaluated by calculating its coefficient of determination (R2). LOD and LOQ values were calculated manually by taking noise to signal ratio of a lowest/known concentration of linearity samples and were expressed in mg/L.
