# Supplementary material for: Optimization of Extraction and HPLC–MS/MS Profiling of Phenolic Compounds from Red Grape Seed Extracts Using Conventional and Deep Eutectic Solvents
Source: Antioxidants (Basel). 2022 Aug 18;11(8):1595. doi: 10.3390/antiox11081595 (PMC9405313; doi:10.3390/antiox11081595)
Supplement: Supplementary file 1 [file antioxidants-11-01595-s001.zip › Table S3.pdf]

Table S3. Results for accuracy (Recovery) and precision (RSD) of HPLC-MS/MS method

	**Ethanol GSEs (n=3)**			**ChCit GSEs (n=3)**		
	Nominal concentration*	Accuracy (Recovery %)	Precision (RSD)	Nominal concentration*	Accuracy (Recovery %)	Precision (RSD)
TA	21.55+0.54	63.60-65.58	1.37	4.95+0.54	70.48-120.82	21.44
	21.55+2.70	81.64-106.74	12.35	4.95+2.70	25.44-31.94	6.39
	21.55+5.40	97.25-102.42	2.46	4.95+5.40	20.51-24.58	5.20
MA	20.10+1.80	75.82-81.05	2.62	15.91+1.80	92.64-98.64	2.36
	20.10+9.00	80.88-83.25	1.18	15.91+9.00	88.80-91.24	1.10
	20.10+18.00	83.01-84.65	0.82	15.91+18.00	71.98-74.27	1.24
GA	10.65+0.18	77.71-79.84	1.35	11.51+0.18	92.44-96.19	2.05
	10.65+0.90	79.73-84-97	3.20	11.51+0.90	97.44-101.43	2.13
	10.65+1.80	85.00-87.60	1.51	11.51+1.80	93.91-96.77	1.56
GC	0.20+0.18	80.13-94.74	18.87	0.23+0.18	80.45-85.59	7.41
	0.20+0.90	82.09-84.43	1.91	0.23+0.90	81.63-82.96	1.07
	0.20+1.80	108.03-110.11	1.05	0.23+1.80	76.49-86.69	7.65
PA	0.050+0.018	64.44-73.73	3.87	0.140+0.018	85.97-102.98	6.35
	0.050+0.090	84.60-105.90	7.26	0.140+0.090	96.72-114.59	7.10
	0.050+0.180	93.09-101.17	3.30	0.140+0.180	99.38-116.41	7.71
PB1	26.30+0.09	75.66-78.41	2.00	39.37+0.09	84.72-88.63	2.49
	26.30+0.45	77.58-81.47	2.75	39.37+0.45	90.06-90.27	0.12
	26.30+0.90	81.91-82.60	0.45	39.37+0.90	82.05-82.57	0.31
EGC	0.29+0.09	78.75-90.03	14.93	0.33+0.09	73.75-85.40	15.84
	0.29+0.45	64.78-74.96	11.51	0.33+0.45	74.66-98.81	18.72
	0.29+0.90	102.56-116.56	8.56	0.33+0.90	89.86-92.18	1.61
C	44.78+1.80	76.45-79.69	1.71	27.58+1.80	70.23-74.79	2.30
	44.78+9.00	88.74-91.51	1.34	27.58+9.00	76.70-79.29	1.49
	44.78+18.00	99.31-103.59	1.93	27.58+18.00	68.87-72.10	1.84
PB2	3.050+0.144	66.07-70.17	2.11	3.050+0.144	86.95-90.62	1.61
	3.050+0.720	79.69-80.98	0.62	3.050+0.720	73.69-83.66	4.85
	3.050+1.440	86.67-97.41	5.54	3.050+1.440	75.69-96.39	9.72
EC	35.59+1.80	89.79-94.63	2.37	14.79+1.80	69.31-75.89	3.00
	35.59+9.00	105.71-110.39	1.85	14.79+9.00	76.97-80.21	1.44
	35.59+18.00	114.10-119.34	1.98	14.79+18.00	71.29-74.33	1.55

*Nominal concentration in spiked samples expressed as determined concentration of analyte in GSEs + known concentration of standard (mg/L)

Legend: RSD- Relative Standard Deviation; GSEs- Grape Seed Extracts; EtOH- ethanol; ChCit- choline chloride:citric acid: TA- Tartaric Acid; MA- Malic Acid; GA- Gallic Acid; GC- Gallocatechin; PA- Protocatechuic Acid: PB1- Procyanidin B1; EGC- Epigallocatechin; C- (+)-Catechin: PB2- Procyanidin B2; EC- (-)-Epicatechin

To evaluate the accuracy and precision of the analytical method, samples were prepared by spiking 50 µl aliquots of concentrated EtOH GSEs extract with 450 µl of each of the first three concentration levels of calibration mix standard solutions, while 100 µl aliquots of concentrated ChCit GSEs extract were spiked with 400 µl of the same mixtures of standard solutions (concentration intervals are given in Supplementary files - Table S2 and S3). Accuracy of the method was reported as determined recovery value measuring related to total spiked concentration. Precision was expressed as relative standard deviation (RSD, %) of the measured concentrations in spiked replicates.
