# Supplementary figures and images for: Optimization of Extraction and HPLC–MS/MS Profiling of Phenolic Compounds from Red Grape Seed Extracts Using Conventional and Deep Eutectic Solvents
Source: Antioxidants (Basel). 2022 Aug 18;11(8):1595. doi: 10.3390/antiox11081595 (PMC9405313; doi:10.3390/antiox11081595)

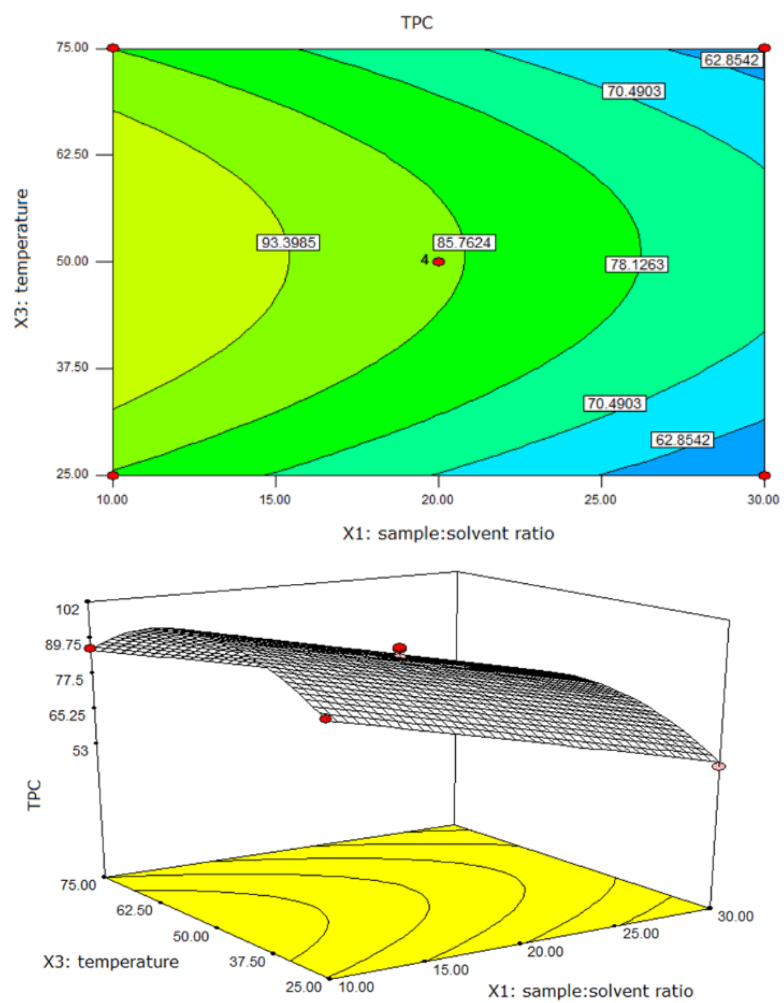

**Figure S1.** Contour plot (A); three-dimensional (3D) response surface (B)

Supplement: Supplementary file 1 [file antioxidants-11-01595-s001.zip › Figure S1.pdf]
